# Emerging role of targeting macrophages in rheumatoid arthritis: Focus on polarization, metabolism and apoptosis

**DOI:** 10.1111/cpr.12854

**Published:** 2020-06-12

**Authors:** Xuezhi Yang, Yan Chang, Wei Wei

**Affiliations:** ^1^ Institute of Clinical Pharmacology Key Laboratory of Anti‐inflammatory and Immune Medicine (Anhui Medical University) Ministry of Education Anhui Collaborative Innovation Center of Anti‐inflammatory and Immune Medicine Anhui Medical University Hefei China

## Abstract

Macrophages maintain a dynamic balance in physiology. Various known or unknown microenvironmental signals influence the polarization, activation and death of macrophages, which creates an imbalance that leads to disease. Rheumatoid arthritis (RA) is characterized by the massive infiltration of a variety of chronic inflammatory cells in synovia. Abundant activated macrophages found in RA synovia are an early hallmark of RA, and the number of these macrophages can be decreased after effective treatment. In RA, the proportion of M1 (pro‐inflammatory macrophages) is higher than that of M2 (anti‐inflammatory macrophages). The increased pro‐inflammatory ability of macrophages is related to their excessive activation and proliferation as well as an enhanced anti‐apoptosis ability. At present, there are no clinical therapies specific to macrophages in RA. Understanding the mechanisms and functional consequences of the heterogeneity of macrophages will aid in confirming their potential role in inflammation development. This review will outline RA‐related macrophage properties (focus on polarization, metabolism and apoptosis) as well as the origin of macrophages. The molecular mechanisms that drive macrophage properties also be elucidated to identify novel therapeutic targets for RA and other autoimmune disease.

## 1 INTRODUCTION

Functionally diverse macrophages play different roles in development and homeostasis. Depending on their location and function in the body, macrophages can be classified into different types such as microglia, Kupffer cells and osteoclasts. In normal physiology, these different macrophages maintain a dynamic balance which, once interfered with, leads to disease.^1^

Rheumatoid arthritis (RA) is an autoimmune disease characterized by synovial inflammation and joint erosion. There are multiple chronic inflammatory cell infiltrations in RA synovium, including synovial macrophages. Macrophages are one of the most abundant cell types in the synovium of RA.^2^ The increased pro‐inflammatory ability of macrophages is related to their excessive activation and proliferation as well as their enhanced anti‐apoptosis ability.^2^ Activated macrophages play an important role in inflammation development by interacting with the inflammatory microenvironment. Macrophages are sensitive to tissue invasion through pattern recognition and phagocytic receptors. When activated, macrophages produce cytokines such as interleukin (IL)‐1β, IL‐6 and tumour necrosis factor (TNF)‐α. These cytokines, in turn, promote inflammation by recruiting additional immune cells, fibroblast activation and T‐cell polarization. The changed microenvironmental factors such as accumulated cytokines, oxidized lipids and other factors in inflammation sites can also influence macrophage activation, polarization and apoptosis. The number of macrophages in RA synovium is significantly correlated with disease activity indicators (including c‐reactive protein level, erythrocyte sedimentation rate, joint swelling count, synovial lining vascular density and thickness, and radiological severity).^3^ Additionally, macrophage depletion can instigate chronic arthritis in both mouse and human systems.^4^ In addition, inflammatory conditions and phosphoinositide 3‐kinase (PI3K) signalling pathways may enhance the anti‐apoptosis ability of macrophages mediated by Fas/Fas ligand interactions or by cytokine withdrawal.^5^ In RA synovium, the anti‐apoptosis ability of pro‐inflammatory macrophages is stronger than that of anti‐inflammatory macrophages.

Multiple studies have demonstrated the potential role of macrophages as a novel therapeutic target in autoimmune disease. In RA, the changes in the number of synovial macrophages and the expression of inflammatory products reflect the therapeutic efficacy.^6^ In addition to numbers, the polarization status of macrophages is closely related to RA. Noticeably, M1 and M2 macrophages appear to be two extreme situations for the dynamically changing macrophage phenotype. Therefore, it is necessary to use the ratio of M1/M2 to evaluate the disease activity and treatment efficacy, rather than the change of a single indicator of M1 or M2. However, an unbiased and systematic approach has not been undertaken to determine the molecular signatures and biological functions of macrophages with dynamically changed phenotypes that underlie the potential targeting of macrophages in RA treatment.

In this review, we discussed our expanding view of RA‐related macrophage properties (focus on polarization, metabolism and apoptosis) as well as the origin of macrophages. The molecular mechanisms that drive macrophage properties also be elucidated to identify novel therapeutic targets for RA and other autoimmune disease.

## 2 THE ORIGIN OF RA‐RELATED MACROPHAGES

**FIGURE 1:**
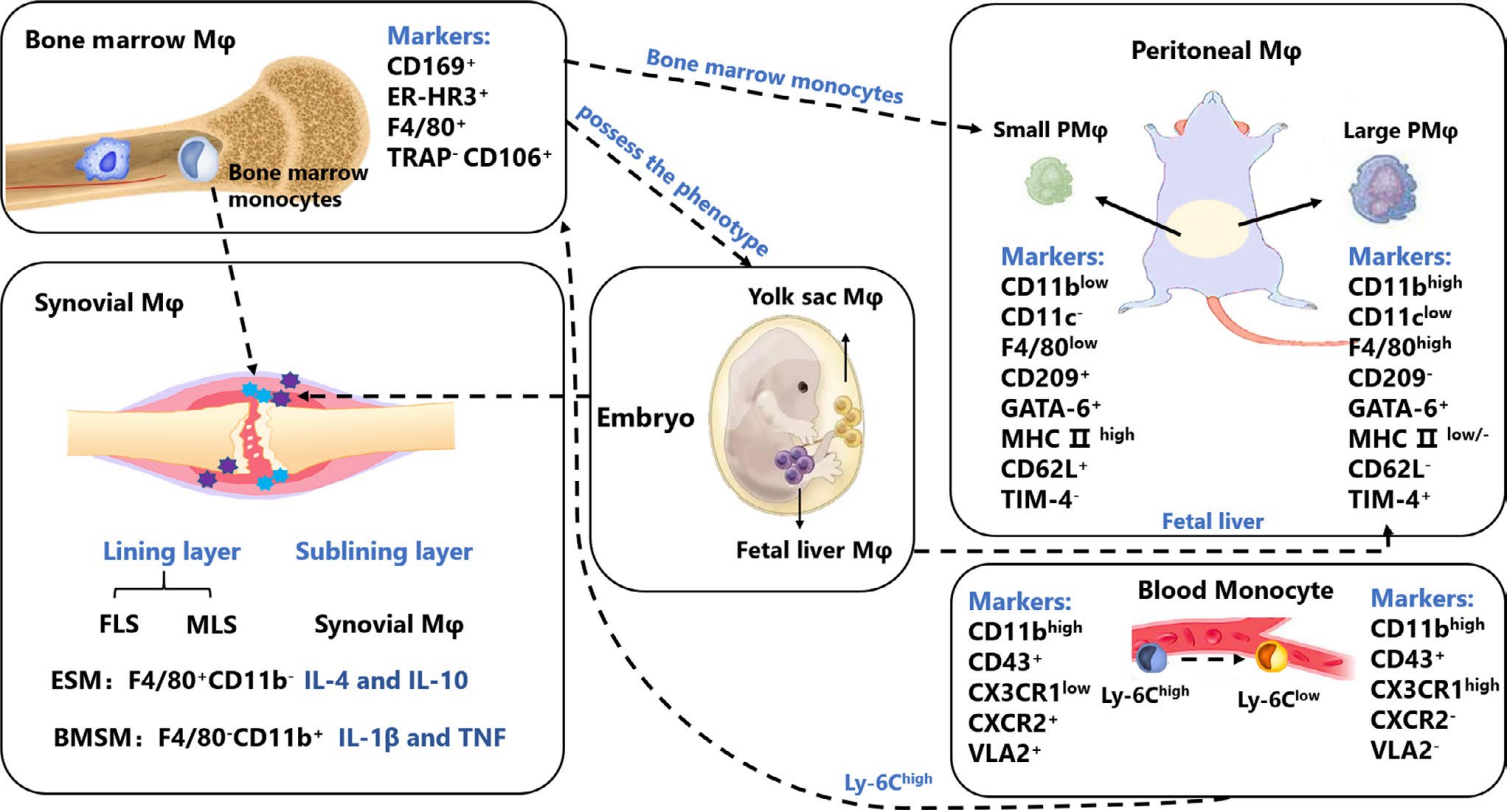
The origin and marks of RA‐related macrophages in mice. Peritoneal macrophages, bone marrow‐derived macrophages, peripheral blood monocytes and synovial macrophages are widely studied in RA and animal arthritis model. These heterogeneous macrophages show different marks, roles and origins. Bone marrow‐derived macrophages are derived from Ly6c^hi^ monocytes, which can possess the phenotype of resident macrophages. Two main macrophage subsets found in peritoneal cavity of adult mice named as LPM and SPM. Approximately 90% peritoneal macrophage are LPMs which are derived from foetal liver macrophages, SPMs are derived from bone marrow‐derived monocytes. Two types of SM were found: embryonic SMs (ESMs) which were F4/80^+^CD11b^‐^ and appeared at a mid‐embryonic stage; and bone marrow‐derived SMs (BMSMs), which were F4/80^‐^CD11b^+^ and appeared at a late‐embryonic stage. ESMs expressed anti‐inflammatory cytokines such as IL‐4 and IL‐10, and BMSMs expressed pro‐inflammatory cytokines such as IL‐1β and TNF

Peritoneal and bone marrow‐derived macrophages are widely studied in arthritis animal models, while peripheral blood monocytes and synovial macrophages are widely studied in patients with RA (Figure 1).

Initially, it was thought that there were two main macrophage subsets found in the peritoneal cavity of adult mice and named as large peritoneal macrophages (LPMs) and small peritoneal macrophages (SPMs).^7^ Approximately 90% of peritoneal macrophages are LPMs which are derived from foetal liver macrophages and disappear rapidly from stimulation by lipopolysaccharide (LPS) or thioglycolate. After the disappearance of LPM, SPM holds the dominant position, but SPMs are derived from bone marrow‐derived monocytes.^7^ More recently, a study has detailed the subtypes of macrophages in the peritoneal cavity of mice.^8^ There are multiple subsets of macrophages in the peritoneal cavity, which can be identified by F4/80, CD64, T‐cell immunoglobulin domain and mucin domain 4 (TIM4), and lymphocyte antigen 6c (Ly6c) staining.^9^ Ly6c^‐^, F4/80^hi^, CD64^+^, and Tim4^‐^, and Ly6c^‐^, F4/80^hi^, CD64^+^, and Tim4^+^ represent resident peritoneal macrophage populations. Tissue‐resident macrophages are mainly derived from yolk sac macrophages and foetal liver monocytes. Recent studies have shown that tissue‐resident macrophages can also be derived from embryos and sustain themselves for a long time through local proliferation independent of hematopoietic stem cells.^10^, ^11^, ^12^ F4/80^lo/‐^, CD64^+^ and Ly6C^+^ represent monocyte‐derived macrophages.^9^

Bone marrow‐derived macrophages are derived from Ly6c^hi^ monocytes, which can possess the phenotype of resident macrophages.^13^ Under normal physiological conditions, tissue‐resident macrophages with a certain proliferation capacity are in a state of low differentiation and low proliferation to maintain homeostasis balance. Under tissue‐resident macrophage depletion, inflammatory conditions or physiological stress, abundant peripheral blood monocytes enter the tissues via blood and differentiate into M1 macrophages with TNF‐α and inducible nitric oxide synthase (iNOS) as the main marker by upregulating their gene expression associated with the macrophage.^14^ The number of peripheral macrophages raised through lymphatic vessels decreases after inflammation.^15^ After the inflammatory response subsides, the bone marrow‐derived macrophages in the liver will replace the resident Kupffer cells.^16^ However, in most cases, the bone marrow‐derived macrophages cannot replace the tissue‐resident macrophages, and most macrophages will undergo programmed death after the inflammatory response subsides.^17^ A small proportion of bone marrow‐derived macrophages will continue to survive, displaying strong plasticity, and can directly differentiate into M2 macrophages. However, this mechanism remains unclear.^18^ In in vitro experiments, macrophage colony‐stimulating factor (M‐CSF) can usually be used to induce monocytes in the bone marrow to differentiate into bone marrow‐derived macrophages.^19^

The normal synovial membrane is comprised of two distinct parts: the intimal lining layer and synovial sublining layer. The intimal lining layer consists of fibroblast synovial cells (FLS) and macrophage‐like synovial cells (MLS). The synovial sublining layer consists of synovial macrophages (SMs), blood vessels and other cells.^20^ Transcriptome analysis of FLS and MLS isolated from patients with RA confirms that MLS is a macrophage with a strong inflammatory tendency.^21^ At first, despite advances in identifying the development of tissue‐resident macrophages, the origins of SMs are elusive. Beige mice carry a gene (bg) that encodes the presence of large intracellular particles in many cell types,^22^ transplanted bone marrow from beige mice into irradiated normal mice. They observed giant granules in the synovial lining cells of bone marrow recipients that were similar to those of the beige mouse, which indicated that bone marrow‐derived cells enter through the synovial lining. Giant granulosa‐bearing cells are also seen beneath the intimal lining layer. Bone marrow recipients were observed by electron microscopy in MLS, and giant granulosa were observed in MLS and FLS in the donor material (beige).^23^ Their research suggests that only MLS originates from bone marrow, possibly because they resemble mononuclear phagocytes elsewhere. However, it is still possible that MLS also originates from the bone marrow, but at a slower rate of replacement. Another study has shown that macrophage populations in the synovial lining may be embryonically derived and bone marrow‐derived through immunotyping,^24^ though further investigations are required to verify this. Recently, the origin of synovial macrophages has been revealed by our group.^25^ Two types of SMs were found: embryonic SMs (ESMs) which were F4/80^+^CD11b^‐^ and appeared at a mid‐embryonic stage; and bone marrow‐derived SMs (BMSMs), which were F4/80^‐^CD11b^+^ and appeared at a late‐embryonic stage. ESMs expressed anti‐inflammatory cytokines such as IL‐4 and IL‐10, and BMSMs expressed pro‐inflammatory cytokines such as IL‐1β and TNF. Recently, Gerhard Krönke et al^26^ found a distinct population of CX3C‐chemokine receptor 1^+^ (CX3CR1^+^) tissue‐resident macrophages at the lining layer, forming an immunological barrier to seclude the joint. CX3CR1^+^ macrophage barrier displays anti‐inflammatory properties and maintains their numbers through locally proliferating CX3CR1^−^ mononuclear cells.

## 3 MACROPHAGE POLARIZATION IN RA

The concept of M1/M2 macrophage polarization is originally proposed to explain the difference in the function of macrophages induced by different external stimuli in vitro. At present, it is believed that macrophages can also be polarized in vivo.^14^

**FIGURE 2:**
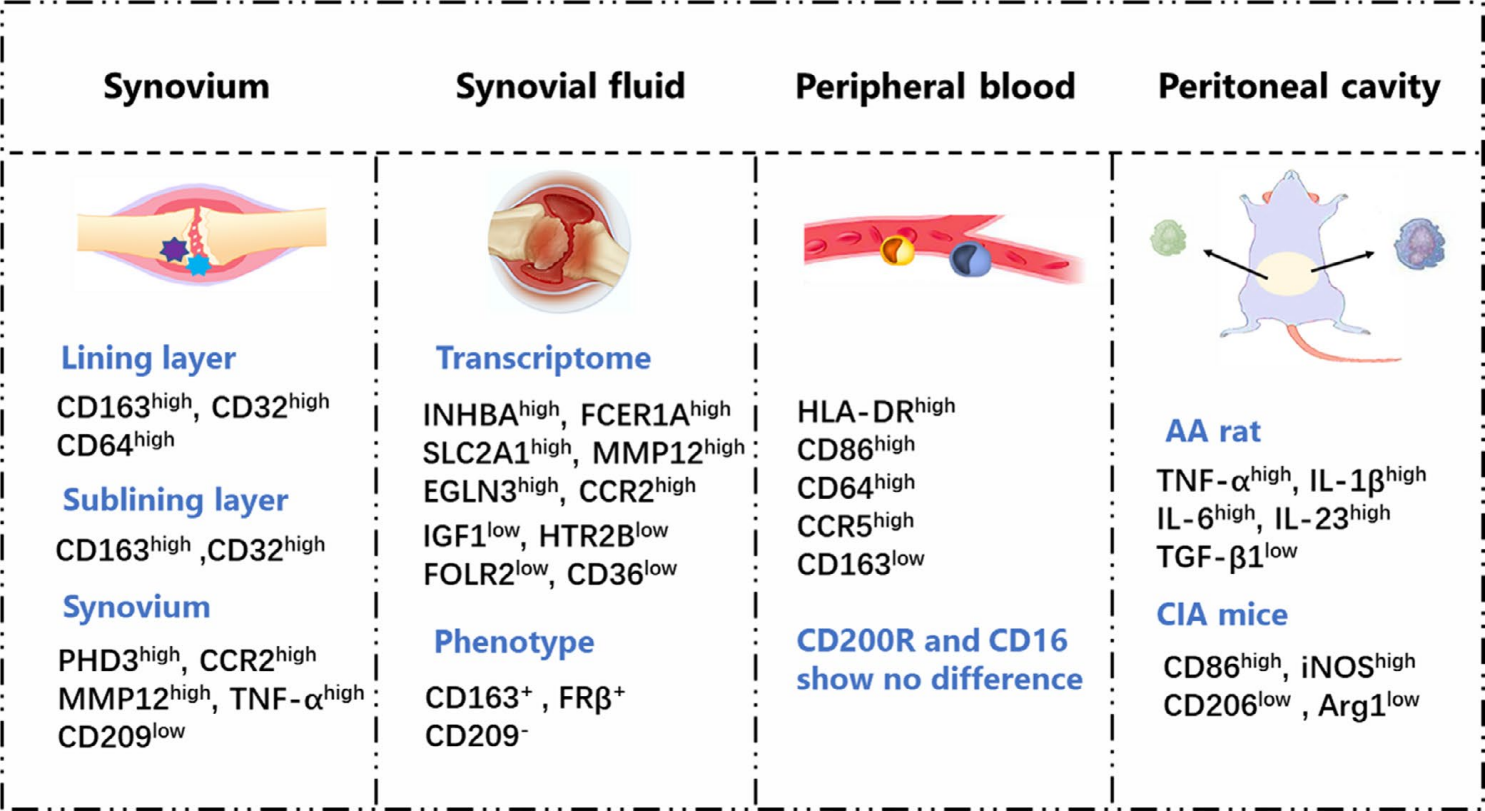
Macrophage polarization in RA. CD163 and CD32 are highly expressed in the synovial lining macrophages of RA patients, CD163, CD32 and CD64 are highly expressed in the macrophages in the lining layer. The synovial macrophages of RA patients highly express MMP12, TNF‐α and the transcription proteins PHD3 and CCR2 of pro‐inflammatory gene EGLN3 and show lower expression of M2 macrophage indicator CD209. RA synovial fluid macrophages express pro‐inflammatory genes (INHBA, FCER1A, SLC2A1, MMP12, EGLN3, CCR2), while express low expression of anti‐inflammatory genes (IGF1, HTR2B, FOLR2, CD36). CD163 and FRβ are expressed in synovial fluid macrophages, while CD209 is not expressed. Some studies confirm that RA synovial fluid highly express M1 macrophage indicators, including HLA‐DR, CD40, CD80, CD86 and CD276. M1 macrophage indicators HLA‐DR, CD86, CD64 and CCR5 are highly expressed in mononuclear macrophages in peripheral blood of RA patients, while M2 macrophage indicators CD163 shows low expression, and CD200R and CD16 show no difference. Peritoneal macrophages from AA rats produce high levels of TNF‐α, IL‐1β, IL‐6 and IL‐23 and low levels of TGF‐β1. Peritoneal macrophages from CIA mice express high levels of CD86 and iNOS and low levels of CD206 and Arg1

Depending on phenotypes and secreted cytokines, macrophages have been classified into many types (Figure 2). However, they are first classified into M1 (inflammatory macrophages) and M2 (anti‐inflammatory macrophages). M2 are further divided into M2a, M2b and M2c for the different stimuli. A study by Mosser et al^27^ have divided macrophages into classically activated macrophages (equal to M1), alternatively activated macrophages (equal to M2a) and regulatory macrophages (equal to M2b/c). In normal physiology, the phenotype of macrophages maintains a dynamic balance. However, the inflammatory immune response is complex and rapid, and the composition of M1/M2 varies at different times, stages of differentiation and tissues^28^ (Table 1).

**TABLE 1:** The subtype, marks and feature of macrophages

	Macrophage	M1	M2				M4	Mox	M(Hb)	Mhem
			M2a	M2b	M2c	M2d				
Markers	CD11a/b/c, CD14/15/16/33/64/32/68/80/85k/86/107b/115/163, CCR5, Mac‐2, GITR Ligand, HLA‐DR, MHCⅡ,TLR2/4, EMR1(Human), F4/80(Mouse)	CD16/32/36/68/80/86^+^,CD163^‐^, IFN‐γR^+^,MHCⅡ^high^; COX2^+^, INOS^+^,IRF5^+^, STAT1^+^	CD163/200/209/206/301^+^,CXCR1/2^+^, Dectin‐1^+^, IL‐1RⅡ^+^, MHCⅡ^low^, Arg1^+^(Mouse), IRF4^+^, PPARγ^+^, STAT6^+^	CD86^+^, IL‐4 Rα^+^, IFN‐γ R^+^, MHCⅡ^+^, COX2^+^, SOCS3^+^, IRF4^+^, Sphingosine Kinase 1/2^+^	CXCR2^+^, CD150/163/206/301^+^, IL‐4 Rα^+^, MSR^+^, SR‐B1^+^, TLR1^+^, Arg1^+^(Mouse), IRF4^+^, SOCS3^+^, TLR8^+^	INOS^+^	Unknown			
Inducer		LPS, IFNγ, TNFα, OXLDL	IL‐4, IL‐13	Immune complex + IL‐1R/TLR ligands	IL‐10, TGF‐β	TLR Agonists + Adenosine	CXCL4	OxPL	Haemoglobin/Haptoglobin Complex	Heme
Feature		Pro‐Inflammatory	Anti‐Inflammatory	Anti‐Inflammatory	Anti‐Inflammatory	Wound Healing& Angiogenesis	Pro‐Inflammatory	Anti‐Oxidant	Anti‐Inflammatory Atheroprotective	

A changed environment stimulates the membrane receptor to start intracellular signal transduction pathways. Activating transcription factors then begin different gene expression. At present, transcription factors involved in macrophage polarization include nuclear factor kappa‐B (NF‐κB), signal transducer and activator of transcription‐1 (STAT‐1), and interferon regulatory factor‐5 (IRF5) in the M1 pathway^29^, ^30^, ^31^ and IRF4, STAT‐6, and peroxisome proliferator‐activated receptor‐γ (PPARγ) in the M2 pathway.^32^, ^33^ Different stimuli activate different transcription factors. Interferon‐γ (IFN‐γ) can phosphorylate STAT‐1,^34^ while IL‐4 and IL‐13 can activate STAT6.^35^ The lack of activated STAT‐1 results in the negative expression of many M1 markers.^31^, ^35^ NF‐κB is the key in M1 polarization, which can regulate the transcription of IL‐1, TNF‐α, IL‐6 and cyclooxygenase‐2 (COX‐2).^29^ IRF is the participating media in macrophage polarization. High level of IRF5 found in M1, involved in the toll‐like receptor (TLR) pathway, inhibits the expression of IL‐10 and promotes Th1‐Th17 responses.^30^, ^36^ IRF1, which has an antagonistic role on IRF4, cooperates with NF‐κB and responds to multiple pro‐inflammatory factors, while IRF4 is key in M2 polarization^32^, ^36^ PPARγ, a type of nucleus receptor, plays an important role in M2 polarization.^33^ The liver X receptor (LXR), which is similar to PPARγ, can also increase the expression of arginase‐1 (Arg1).^37^

### 3.1 Macrophage polarization in RA synovium

Mass immune cells, such as macrophages and lymphocytes, are observed in both the lining and sublining layer of patients with RA. A previous study has shown that the macrophage polarization in these two layers is different. Ambarus et al^38^ found that M2 macrophage indicators CD163 and CD32 are highly expressed in the synovial lining of macrophages of patients with RA, indicating that M2 macrophages are predominant in the sublining layer. CD163, CD32 and M1 macrophage indicator CD64 are highly expressed in the lining layer of the macrophages, indicating that these macrophages are a mix of M1 and M2 macrophages. However, compared with normal synovial tissues, the synovial macrophages of patients with RA highly express matrix metalloproteinase‐12 (MMP12), TNF‐α and the transcription proteins PHD3 and CCR2 of the pro‐inflammatory gene EGLN3 and show a lower expression of the M2 macrophage indicator CD209.^39^ These abnormal indicators suggest that M1 macrophages are dominant in the RA synovium (Figure 2).

### 3.2 Macrophage polarization in RA synovial fluid

Soler et al^39^ discuss the macrophage polarization in RA synovial fluid through hierarchical clustering analysis and other means. In terms of the transcriptome, RA synovial fluid macrophages express pro‐inflammatory genes (INHBA, FCER1A, SLC2A1, MMP12, EGLN3 and CCR2), while exhibiting a low expression of anti‐inflammatory genes (IGF1, HTR2B, FOLR2 and CD36). In terms of phenotypes, CD163 and FRβ are expressed in synovial fluid macrophages, while CD209 is not expressed. Some studies confirm highly expressed M1 macrophage indicators including HLA‐DR, CD40, CD80, CD86 and CD276.^40^ In addition, Zhu et al^41^ indicate that the M1/M2 ratio of the synovial fluid macrophages in patients with RA significantly increases to 32.76 ± 11.02. These results confirm that M1 macrophages are dominant in RA synovial fluid (Figure 2).

### 3.3 Macrophage polarization in peripheral blood of RA patients and peritoneal cavity of arthritis model

Some highly expressed pro‐inflammatory factors in patients with RA may induce the monocytes which differentiate into M1 macrophages during the migration to synovial tissues. M1 macrophage indicators HLA‐DR, CD86, CD64 and CCR5 are highly expressed in mononuclear macrophages in the peripheral blood of patients with RA, while M2 macrophage indicator CD163 shows low expression, and CD200R and CD16 show no difference,^38^ indicating that the mononuclear macrophages in the peripheral blood of patients with RA tend to be M1 macrophages (Figure 2).

Our previous study found that peritoneal macrophages from AA rats produce high levels of TNF‐α, IL‐1β, IL‐6 and IL‐23 and low levels of transforming growth factor‐β1 (TGF‐β1).^42^ Peritoneal macrophages from CIA mice express high levels of CD86 and iNOS and low levels of CD206 and Arg1^43^ (Figure 2).

## 4 THE SPECIFIC ROLE OF MACROPHAGES IN RA

**FIGURE 3:**
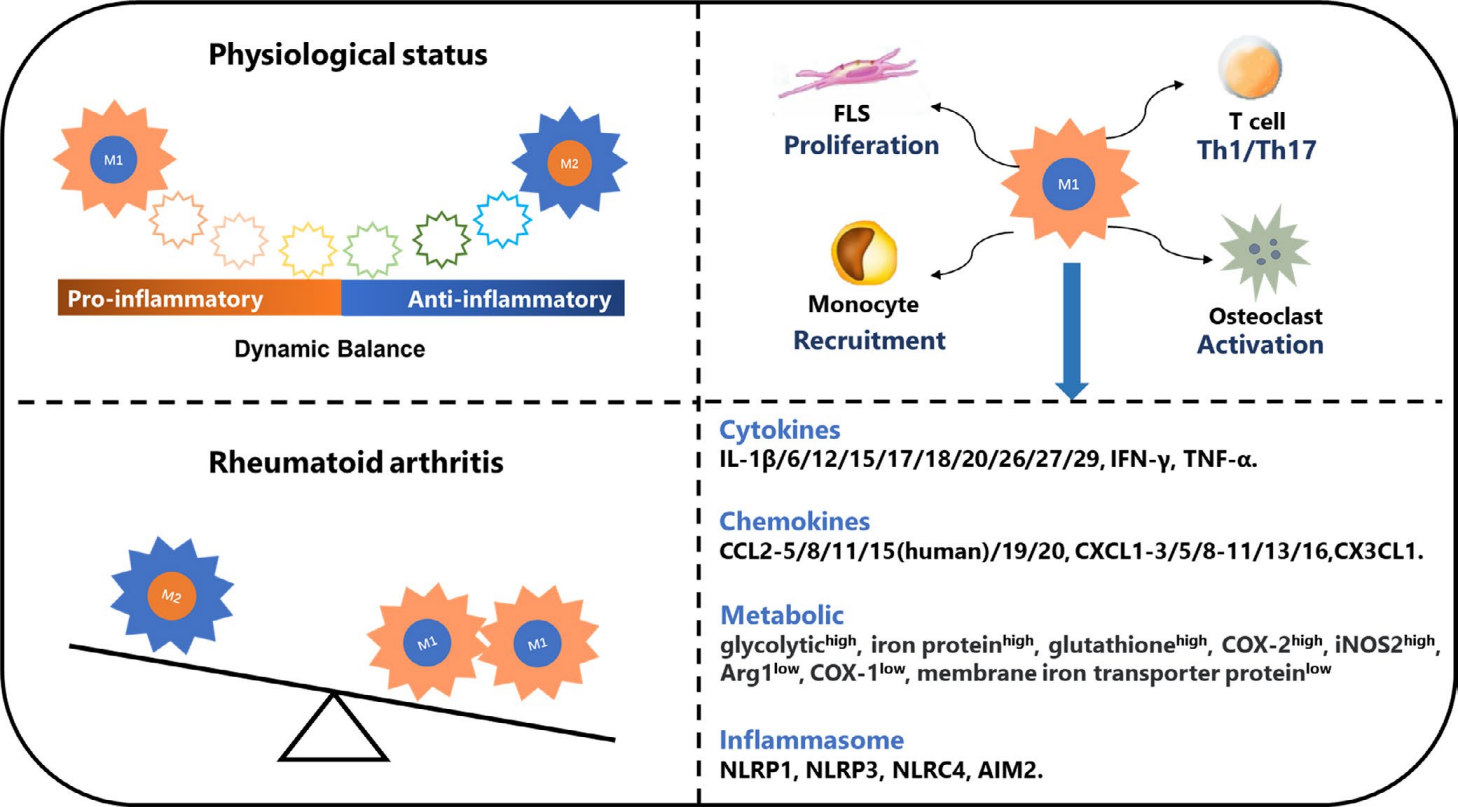
The specific roles of macrophages in RA. M1 and M2 seems like two extreme situations and the phenotypes of macrophages keep a dynamic balance in physiological status. In general, M1 is more than M2 in RA patients and arthritis animal models. There are multiple chronic inflammatory cell infiltrations in RA synovium, and macrophages can secret various factors and interact other cells to involved in pathogenesis. Macrophages induce FLS proliferation and activation through producing IL‐1β and TNF. Activated FLSs secret RANKL and M‐CSF, resulting in osteoclast formation and activation, which is also induced by macrophage‐secreted IL‐1β, IL‐6 and TNF. Macrophages enable monocytes recruitment in RA synovium through producing IL‐1β and TNF. The T‐cell differentiation play important roles in RA process, macrophages‐secreted IL‐23 activates Th17 cells, and macrophage‐secreted IL‐12 and TNF activate Th1 cells

Macrophages interact with a microenvironment to participate in RA inflammation development (Figure 3). When activated, macrophages produce cytokines, chemokines, metabolites and other factors to participate in the RA process.

### 4.1 Macrophage mediator in RA

Mediators secreted by macrophages have been extensively summarized. Here, we focus on mediator newcomers secreted by macrophages in RA.

M1 macrophages in synovium can secret chemokine C‐X‐C motif ligand (CXCL) to promote inflammation and angiogenesis from CXCL1 to CXCL10.^44^ Elevated levels of CXCL8 have been detected in the synovial fluid and serum of patients with RA and are associated with clinical manifestations of RA.^44^, ^45^ The high levels of CXCL16 found in RA synovium are involved in monocyte recruitment into the RA synovial tissue.^46^ Chemokine (C‐C motif) ligand 2 (CCL2), CCL3 and CCL5 drive neutrophil infiltration into the joints of adjuvant arthritis (AA) rats.^45^ The inhibition of CCL2 can ameliorate AA rats.^45^

IL‐1, IL‐6 and TNF‐α as the primary pro‐inflammatory cytokines in RA are mainly secreted by synovial macrophages in RA. IL‐12, IL‐15 and IL‐18 contribute to the differentiation of T helper type 1 (Th1), and IL‐18 is reported to induce the production of CCL2.^46^ Immunohistochemistry reveals that FLS and MLS are the main IL‐26‐producing cells in RA joints. IL‐20, mainly produced by macrophages, is independently associated with RA disease activity and may be triggered by TLR ligands at local sites of inflammation.^47^ IL‐22, produced by FLS and macrophages, promotes inflammatory responses in RA synovial tissues by inducing FLS proliferation and chemokine production.^48^ IL‐26 induces pro‐inflammatory cytokines and Th17 cell generation.^49^ IL‐27 is expressed in synovial macrophages, and increased levels of IL‐27 relieve arthritis in CIA mouse ankles. This amelioration of arthritis involves a reduction in CIA mouse serum and joint levels of IL‐17.^50^ IL‐29 is expressed predominately in synovial macrophages and fibroblasts. Increased IL‐29 levels are detected in RA synovial fluid when compared with osteoarthritis synovial fluid, which enhances synovial inflammation and cartilage degradation.^51^

Macrophage migration inhibitory factor (MIF), primarily secreted by macrophages and monocytes, can promote cell survival and growth. Yoo et al^52^ have demonstrated that high expression of MIF alleles (rs5844572) is associated with RA joint erosion. The antagonism or absence of MIF can alleviate arthritis in the animal model of RA, and MIF serum level is positively associated with RA severity.^53^ In addition, MIF can increase the expression of TNF‐α, IL‐1, IL‐6, CXCL8 and MMP‐2 in RA. Large amounts of MMP‐1, MMP‐2, MMP‐9 and MMP‐13 secreted by macrophages are involved in the disruption of the cartilage and bone in RA.^54^ CCL5 can activate the expression of MMP‐1 and MMP‐13 to induce collagen degradation.^54^ In addition to the media described above, macrophages produce some other mediums which are not discussed in detail.

### 4.2 Macrophage metabolism in RA

Cells have two different sources of energy supply: one comes from aerobic metabolism with high‐efficiency, while the other is less efficient but faster glycolysis. Glycolysis does not require oxygen participation and is often triggered by inflammatory stress. Macrophage polarization involves a coordinated metabolic process that is only partially understood. The main features of M1 metabolism include the high expression of a glycolytic enzyme, iron protein, glutathione, COX‐2, and the high activity of iNOS2, and low expression of a membrane iron transporter protein, COX‐1, and the low activity of Arg1. The main features of M2, which show a high level of oxidation of fatty acids, is the opposite M1^55^, ^56^, ^57^, ^58^ (Figure 3).

The macrophages of M1 and M2 are, respectively, in a state of reduction and oxidation. One of the important features of M1 macrophages is the production of reactive oxygen species (ROS). RA macrophages are overwhelmed by overnutrition and become victims of excessive glucose uptake, producing high levels of ATP and mitochondrial ROS.^59^ High levels of ROS participate in the destruction of joints and cartilage in RA.^59^ LPS can decrease the levels of oxidative phosphorylation (OXPHOS) and enhance the levels of glycolysis in macrophages.^56^ LPS‐induced glycolysis results in the accumulation of an intermediate metabolite of the tricarboxylic acid cycle, especially succinic acid.^57^ Succinic acid can combine with the GPR91 of itself and other macrophages and trigger them to release IL‐1β.^57^ High levels of succinic acid are found in the synovial fluid of patients with RA, and mice macrophages cultured in this synovial fluid secret a high level of IL‐1β.^57^ A previous study has identified increased markers of hypoxia and glycolytic metabolites in RA synovial fluid.^55^ Elevated hypoxia levels were found to be negatively correlated with increased synovial inflammation.^55^ Oxidative stress is conducive to glycolysis, which helps to accelerate the occurrence of inflammation and subsequent angiogenesis dysfunction in RA.^60^ Abboud et al^61^ found that inhibiting glycolysis reduces the disease severity in a K/BxN mouse model, which is a spontaneous model of arthritis driven by T‐cell receptor transgenic CD4^+^ T cells.

Iron has been studied in depth with regard to the interconnection of iron homeostasis with the biology of M1 and M2 macrophages.^62^ M1 macrophages limited the availability of iron essential for circulating pathogens by inducing small changes in the extracellular iron flux. M2 macrophages are highly specialized for iron recycling from senescent erythrocytes via erythrophagocytosis, producing about 90% of the iron needed for erythrocyte production. Anaemia caused by inflammation or chronic diseases is the most common disease‐related complication in patients with RA.^63^ Anaemia in RA can cause severe symptoms and aggravate the manifestations of other diseases. Early detection of anaemia is crucial and treating imbalances of iron may be a new approach for treating RA.

Studies on lipids have confirmed that lipid metabolism is related to macrophage activation. In macrophages, rich endoplasmic omentum and free cholesterol promote the esterification reaction of cholesterol acyltransferase‐1, leading to more free cholesterol and increased inflammatory signalling, especially NF‐κB and TLR signals.^64^ Hofkens et al^65^ have demonstrated that intravenously delivered glucocorticoid liposomes inhibit osteoclast activity and bone erosion in AA mice. Wendt et al^66^ have revealed that lipid metabolism in AA rats presents a strong catabolic tendency, a condition that may lead to marked cachexia in AA rats, or severe RA. Arachidonic acid (ARA) pathway can synthesize pro‐lipid regulation agents in inflammatory reactions, such as prostaglandin E2 (PGE2). In AA rats, the expression of PGE2 is increased in macrophages and FLS. The constant stimulation of PGE2 results in the over‐desensitization of EP4, and the loss of the role of PGE2 in normal physiology.^67^ In addition, ARA can combine with LXR to inhibit TLR4‐activated M2 polarization.^68^

Various amino acids in the metabolic pathway of macrophages also play important roles in RA. In macrophages, the intracellular metabolism of l‐arginine (l‐Arg) is mainly regulated by two enzymes: iNOS and Arg1. iNOS is catalysed by l‐Arg into NO and l‐citrulline. l‐citrulline has been realized as a biomarker in RA. Anti‐cyclic citrullinated peptide (ACCP) antibodies can be recommended for the early detection of RA, decreasing joint damage and deformity.^69^

Glutamine (Gln) is the main metabolic substrate of mononucleate macrophages, which provides energy for cell metabolism through glutamine fermentation.^70^ Gln can affect the phagocytic function of macrophages by changing the content of ATP. Spiter et al^71^ showed that low concentrations of Gln can down‐regulate the expression of cell surface receptors, especially the expression of high‐affinity IgG (FCrRI/CD64) and CR3 (CD11b/CD18), leading to decreased ability of phagocytic sensitized IgG. Wallace et al^72^ found Gln‐deficient cultures, IL‐1 secretion dropped by 60%, suggesting that the ability of macrophages to synthesize and secrete IL‐1 is Gln‐dependent. Similarly, Moskovitz et al^73^ also observed that Gln is necessary for the synthesis of TNF‐complex and IL‐1 in the final differentiation stage of macrophages. The accumulation of Gln activates the mechanistic target of rapamycin (mTOR).^74^ The approaches targeting the inhibition of mTOR appear to benefit patients with RA.^75^

### 4.3 Macrophage death in RA

The death of activated macrophages has been implicated in the pathogenesis of RA. Reportedly, the anti‐apoptosis ability of pro‐inflammatory macrophages is higher than that of anti‐inflammatory macrophages in the synovial fluid of patients with RA.^60^ Enhancing the anti‐apoptosis ability or increasing the anti‐inflammatory macrophage population is a potential treatment for RA.

Apoptosis is a widely accepted procedure of cell death which depends on the activation of cysteine aspartate‐specific protease (caspase) caspase‐3, caspase‐8 and caspase‐9. Cell pyroptosis is a method of programmed cell death which causes the release of cellular content and activates a strong inflammatory response. It is characterized by the dependence of caspase‐1 and caspase‐11, and the release of abundant inflammatory cytokines.^76^ However, the molecular signatures defining the death resistance of RA macrophages are not fully understood.

Macrophage persistence at the inflammatory site likely contributes to RA pathology. The persistent activation of NF‐κB, STAT3 and PI3K signalling pathways in macrophages enhances the resistance to apoptosis and offers better survival conditions to macrophages in the inflammatory RA synovium.^77^

Inflammasomes related to caspase‐1 and caspase‐11 activation contribute to macrophage pyroptosis, the secretion of IL‐1β and IL‐18, and the promotion of Th1 and Th17 differentiation.^77^ There are four core types of inflammasomes: pyrin domain containing 1 (NLRP1), NLRP3, CARD domain containing 4 (NLRC4) and absent in melanoma 2 (AIM2). In addition, NLRP3 and AIM2 activate caspase‐8 and participate in apoptosis induction. NLRP3 is one of the most extensive inflammasomes which have been studied in depth. The abnormal activation and regulation of NLRP3 play an important role in RA. NLRP3 regulation can alleviate disease processes to different degrees. For example, the TNFAIP3/A20 locus has been implicated as a positively associated factor in RA. The arthritis of A20myel‐KO mice depends on the NLRP3 inflammasome and IL‐1R signals. The lack of A20 in macrophages significantly enhances NLRP3‐mediated caspase‐1 activation, pyroptosis and IL‐1β secretion.^78^ The deletion of NLRP3 and caspase‐1 improves RA‐associated inflammation and cartilage destruction in A20myel‐KO mice.

## 5 THE DEVELOPMENT OF ANTIRHEUMATIC DRUGS

Current treatments for RA involve DMARDs, NSAIDs, biologicals, glucocorticoids and botanical agents (Table 2). No drugs are specific and safe for macrophages under clinical conditions, but they do inhibit some effects of macrophage activation, such as the production of inflammatory macrophage cytokines including TNFα, IL‐1 and IL‐6. Inhibition with antibodies or soluble receptors has been used for patients with RA for many years. Novel agents that target these factors are more efficacious in RA. Tocilizumab is the first fully human monoclonal antibody to target IL‐6 receptors directly and was approved for treatment in patients with active moderate to severe RA in 2010.^79^ Adalimumab and tocilizumab also impact on the hepcidin‐mediated alteration of iron homeostasis.^80^ Jain et al encapsulated the IL‐10 encoding plasmid DNA into non‐condensing alginate‐based nanoparticles to transfect the macrophages of arthritic rats. This treatment significantly reduced the expression of TNF‐α, IL‐1β, and IL‐6 and ameliorated joint damage.^81^ The Janus kinase (JAK)‐STAT pathway is a common signalling pathway for multiple cytokines and their receptors participating in the biological processes of macrophages. However, the complete inhibition of cytokine activity also harms the cells physiological functions.^82^ JAK1‐mediated IFN and IL‐6 signalling likely play a key role in the synovial response. Tofacitinib and JAK inhibitors have been reported to reduce MMP and IFN gene expression in RA, and clinical improvements correlate with reductions in STAT1 and STAT3 phosphorylation.^82^

**TABLE 2:** Novel macrophage‐related therapeutic agents for RA

Agents	Immune target	Development phase	Role on macrophages
H22 (scFv)‐MAP^89^	CD64	Preclinical	CD64^+^ M1 macrophage apoptosis
MOR103^87^	GM‐CSF	Clinical Phase II	Inhibition of M1 polarization
Mavrilimumab^88^	GM‐CSF receptor α	Clinical Phase II	Inhibition of M1 polarization
CC‐292^91^	BTK	Clinical Phase II	Inhibition of macrophage‐secreted nitric oxide, TNF‐α and IL‐Iβ
Tofacitinib^82^	JAK1/JAK3	Approved	Blockade of macrophage‐secreted inflammatory factors
Etanercept^24^	TNF	Approved	Increase macrophage apoptosis
Tocilizumab^79^	IL‐6 receptor	Approved	Inhibition of monocyte IL‐6 mRNA expression
Anakinra^24^	IL‐1 receptor	Approved	Blockade of IL‐1 cytokine
Ustekinumab^96^	IL‐12/IL‐23	Approved	Blockade of IL‐12/IL‐23 cytokine
Clodronate liposomes^97^	Release chlorophosphate	Open study in RA patients	Deplete synovial macrophages

The specific strategy on macrophages in RA can depend on the related transcriptional factors, metabolites and factors involved in macrophage death, to change the pro‐inflammatory monocyte and macrophage phenotype or decrease the resistance to death of inflammatory macrophages.

Some newly discovered interleukins can also indirectly regulate the function of macrophages. Targeting cytokine secretion‐associated RA imbalance may be a reliable method to control the disease. IL‐34 promotes monocyte survival, proliferation and differentiation to macrophages.^83^ IL‐35 promotes TNF‐α‐induced apoptosis of FLS and stimulates M2 macrophages polarization, thus inhibiting CIA inflammation.^84^ IL‐37 expression increased in tuberculosis patients promoted M2 macrophages polarization.^85^ As the innate immune inhibitor, IL‐37 reduces joint inflammation of arthritis animal model. IL‐38 overexpression induces the anti‐inflammatory effects in arthritis animal models and in THP‐1 in vitro. In addition, the deletion of IL‐38 in mice exacerbated arthritis.^86^

Granulocyte‐macrophage colony‐stimulating factor (GM‐CSF) is a pro‐inflammatory cytokine that can induce monocyte differentiation to M1 in inflammatory sites of RA. In CIA, treatment with an anti‐GM‐CSF‐receptor antibody resulted in the reduction of clinical arthritis scores and decreased F4/80^+^ macrophages in the synovium.^87^ In addition, mavrilimumab is a monoclonal antibody against the human GM‐CSF receptor alpha chain. Phase II trial outcomes of mavrilimumab have shown promising results for RA in patients.^88^

In patients with RA, hyperplasia macrophages in synovium express high levels of CD64, and a CD64‐directed immunotoxin promotes their selective elimination via apoptotic cell death.^43^ In patients with RA, hyperplasia macrophages in synovium express high levels of CD64, and a CD64‐directed immunotoxin promotes their selective elimination via apoptotic cell death.^43^ New human cytolytic fusion proteins (hCFPs), namely the H22 (scFv)‐MAP, can induce CD64^+^ M1 macrophage apoptosis in mice arthritis models, transforming M1 into M2 macrophages, exerting anti‐inflammatory effects.^89^

Under the activation of TLR4 and NF‐κB in macrophages, nuclear factors of activated T‐cell 5 (NFAT5) promote the expression of pro‐inflammatory markers.^90^ Choi et al found that in RA synovial macrophages, increased NFAT5 expression promotes the anti‐apoptotic ability of macrophages by producing CCL2. In addition, NFAT5 expression in M1 macrophages is higher than that in M2 macrophages.^90^

As an important intracellular kinase, Bruton's tyrosine kinase (BTK) is being investigated for RA treatment. BTK genes mutations are associated with X‐linked immunodeficiency in mice and macrophages from these mice produce less nitric oxide, TNF‐α and IL‐Iβ.^91^ CC‐292 is the first irreversible BTK inhibitor that has been evaluated in a Phase II study. Forty‐seven patients with active RA who had an inadequate response to methotrexate were randomized to receive either CC‐292 375 mg daily or a placebo. The primary endpoint is the ACR20 response at four weeks.^92^

Macrophage‐derived microvesicle‐coated nanoparticles (MNPs) are developed to target RA. This treatment is inspired by the intrinsic inflammation‐targeting capacity of macrophages. Tacrolimus is encapsulated in MNP and significantly suppresses the progression of CIA through targeting Mac‐1 and CD44.^93^

Human umbilical cord blood mesenchymal stem cells inhibit the formation of M1 macrophages in arthritic mice and meanwhile promote the generation of M2 macrophages through TNF‐mediated COX‐2 and TSG‐6 pathways.^94^

## 6 CONCLUSION

Macrophages are endowed with considerable plastically, offering many targets for the development of new therapies to treat disease. Macrophages are one of the most abundant cell types in the synovium of RA. The increased pro‐inflammatory ability of macrophages relates to their excessive activation and proliferation as well as their enhanced anti‐apoptosis ability. The origin, polarization and activation of heterogeneous macrophages have been studied widely. However, the origin of synovial macrophages is currently unknown. The infiltrated or tissue‐resident hyperplasia macrophages found in synovium still need to be investigated. The current classification system does not reflect the diversity of macrophage activation and new subtypes of macrophages might exist. Activated macrophages play an important role in inflammation development through interactions with the inflammatory microenvironment. In RA synovium, multiple numbers of immune cells and non‐immune cells interact, generating a complex inflammatory network. The development of effective and safe therapies is paramount to disease treatment. The inhibition of the pathogenic effects of cytokines is often attempted in the development of therapeutic drugs to treat RA. However, the resulting serious adverse reactions are closely related to the destruction of the physiological functions and network balance of cytokines. For example, IL‐6 can stimulate the proliferation of FLS, enhance the osteoclast activity and lead to pannus formation. IL‐6 and IL‐1 work together to increase matrix metalloproteinase production, leading to the destruction of articular cartilage. However, the activity of IL‐6 and its receptor signals being completely inhibited may lead to a weakened immune system, bone marrow suppression and an increased risk of infection and tumours. The JAK‐STAT pathway is a common signalling pathway of multiple cytokines and their receptors, which is involved in many important biological processes such as cell proliferation, differentiation and apoptosis. The inactivated mutation of JAK3 can lead to immune deficiency. JAK3 is the intracellular molecule necessary for IL‐2, IL‐4, IL‐7, IL‐9, IL‐15, IL‐21 and other cytokines to maintain normal immune cell development, hematopoietic cell survival signal and immunoglobulin class conversion.^74^ Tofacitinib, a JAK3 inhibitor for the treatment of RA, inhibits multiple inflammatory factors through a common target, but the inhibition of the activity also harms cell physiological functions. The number of NK cells and CD8^+^ T cells decrease in patients treated with tofacitinib, and the most common severe adverse reactions are upper respiratory tract infection and opportunistic infection. The incidence of a malignant tumour is similar to that of other biological drugs reported previously. Therefore, it is proposed that the soft regulation of inflammatory immune responses (SRIIR) with regard to the balance of regulation on the activity of common key molecules in multiple target cells is an important direction for treating inflammatory immune responses.^95^

Considering the heterogeneity and dynamically changed phenotypes of macrophages, especially the difference between ESM and BSM in RA, targeting macrophages in RA is suitable for regulating their balance in terms of number, polarization, metabolism and death. For instance, incapacitating or reducing the pro‐inflammatory macrophage population, enhancing the anti‐apoptosis ability, increasing the anti‐inflammatory macrophage population, targeting the activity of common key molecules in macrophages and regulating the M1/M2 ratio in RA back to normal physiology may all be profound in the treatment of RA. In addition, macrophages are natural phagocytes; the cell‐derived membrane structures of extracellular vesicles (EVs) allow intercellular communication. Reportedly, EVs from mesenchymal cells also showed immunosuppression in RA by inhibiting M2 polarization. Altogether, targeting physiological macrophages in RA will lead to the development of RA precision treatment.

## CONFLICT OF INTERESTS

The authors declare that there is no conflict of interests.

## AUTHOR CONTRIBUTIONS

Study concept and design: WW, YC, and. XY; Writing of the paper: XY, YC and WW; All authors reviewed the manuscript prior to submission.

## Data Availability

The data that support the findings of this study are available from the corresponding author upon reasonable request.
